# Interactions of Root-Feeding Insects with Fungal and Oomycete Plant Pathogens

**DOI:** 10.3389/fpls.2017.01764

**Published:** 2017-10-20

**Authors:** Telsa Willsey, Syama Chatterton, Héctor Cárcamo

**Affiliations:** ^1^Department of Biology, University of Lethbridge, Lethbridge, AB, Canada; ^2^Lethbridge Research and Development Centre, Agriculture and Agri-Food Canada, Lethbridge, AB, Canada

**Keywords:** fungi, oomycetes, pathogen–insect interactions, direct interactions, indirect interactions, phytopathogens, root-feeding insects, tripartite interactions

## Abstract

Soilborne fungal and oomycete pathogens are the causal agents of several important plant diseases. Infection frequently co-occurs with herbivory by root-feeding insects, facilitating tripartite interactions that modify plant performance and mortality. In an agricultural context, interactions between pathogens, herbivores, and plants can have important consequences for yield protection. However, belowground interactions are inherently difficult to observe and are often overlooked. Here, we review the impact of direct and indirect interactions between root-associated insects, fungi, and oomycetes on the development of plant disease. We explore the relationship between insect feeding injury and pathogen infection, as well as the role of insects as vectors of fungal and oomycete pathogens. Synergistic interactions between insects and phytopathogens may be important in weed suppression, and we highlight several promising candidates for biocontrol. Bridging the gap between entomological and pathological research is a critical step in understanding how interactions between insects and microorganisms modify the community structure of the rhizosphere, and how this impacts plant functioning. Furthermore, the identification of belowground interactions is required to develop effective pest monitoring and management strategies.

## Introduction

**FIGURE 1:**
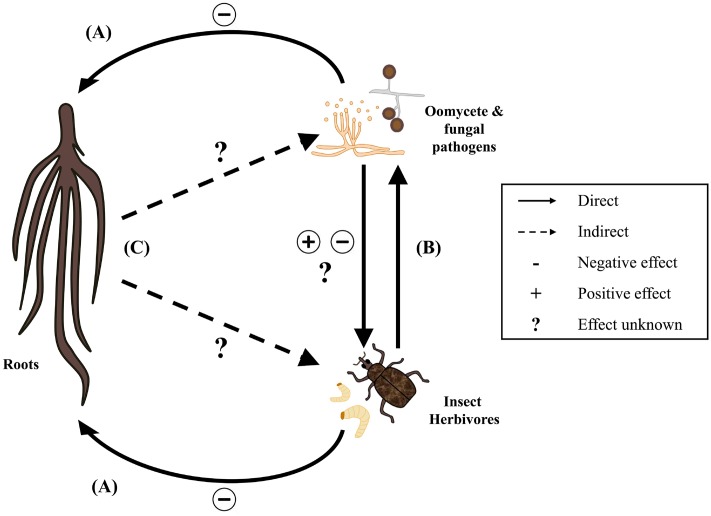
Direct and indirect interactions of plants, root-feeding insects, and oomycete and fungal plant pathogens. Insect herbivores and phytopathogens have direct negative effects on plant performance **(A)**. When attacking a mutual host, insects and pathogens may also influence each other directly with positive, negative, or unknown effects **(B)**. Plants may mediate indirect interactions between insects and pathogens through physical or physiological responses to root damage **(C)**. The underlying mechanisms of plant-mediated interactions and impacts on the community structures of root-associated insects, fungi, and oomycetes are largely unknown.

Phytopathogenic fungi and oomycetes cause many highly destructive plant diseases, often with severe economic consequences for producers (Meng et al., 2009). While they are taxonomically distinct, mechanisms of pathogenesis are often similar between the two groups due to shared morphological and physiological traits (Meng et al., 2009). When microbial infection and insect herbivory occur at the same time, interspecific interactions that alter the individual effects of either organism on plant performance can occur. These interactions may take place directly or indirectly (**Figures 1A–C**) (Hatcher, 1995). Direct interactions occur when the ability of one organism to access plant resources is altered by another, without any influence from the plant itself (**Figure 1B**). Indirect interactions include those in which one organism induces a physical or physiological change in the plant that modifies its response to other organisms (**Figure 1C**). Indirect interactions are therefore plant-mediated. In either case, tripartite interactions between plants, pathogens and insects can complicate diagnoses and make infestation patterns and yield loss difficult to predict (Pieterse and Dicke, 2007). Despite growing recognition of the relationship between plant-associated insects and fungal or oomycete pathogens (see Hatcher, 1995; Hatcher and Ayres, 1997; Fournier et al., 2006; Stout et al., 2006), our understanding of the interface between entomological and pathological research remains limited. Moreover, existing research is heavily biased toward insects and pathogens that attack aerial parts of the plant. Far less is known about how these organisms interact belowground, as subterranean interactions are difficult to observe. As a result, there is a substantial gap in our understanding of how interspecific interactions between soilborne plant pathogens and root-feeding insects can impact pest population dynamics and plant development. The identification of belowground interactions is essential, both to our understanding of rhizosphere ecology and to inform effective management strategies in agricultural crops. The purpose of this review is to discuss currently available information regarding direct and indirect interactions between root-feeding insects, fungal and oomycete pathogens, and plants. In doing so, major gaps in our understanding of these interactions will be identified as potential areas for future research. Only interactions involving phytopathogenic microbes will be considered, as the association of root-feeding insects with plant mutualists and symbionts has been reviewed in detail elsewhere (Johnson and Rasmann, 2015).

## Direct Interactions

Root-associated insects and phytopathogens often attack plants simultaneously, inevitably leading to direct insect–microbe interactions. Currently, there are two main avenues of research regarding direct interactions of rhizophagous insects with soilborne fungal and oomycete plant pathogens: (1) pathogen colonization of insect feeding injury, and (2) insects as vectors of root pathogens. As a result, there are several well-established examples of insects that facilitate the development of root diseases, and these will be discussed in more detail below. Less is known about how fungi and oomycetes directly impact the population dynamics of insects in soil, despite evidence that aboveground pathogens can influence insect development and mortality. For example, the European grapevine moth *Lobesia botrana* Den. & Schiff. (Lepidotera: Tortricidae) interacts mutualistically with the fungal pathogen *Botrytis cinerea* Pers. (Mondy et al., 1998). Larvae act as vectors of *B. cinerea*, and ingestion of the fungus increases the survival and development of larvae and the fecundity of adult insects (Fermaud and Le Menn, 1992; Mondy et al., 1998; Mondy and Corio-Costet, 2004). Evidence of similar interactions occurring below the soil is largely lacking. Clover root borers (*Hylastinus obscurus* Marsham; Coleoptera: Scolytidae) show a clear preference for red clover roots infected with fungal pathogens over healthy roots (Leath and Byers, 1973). Additionally, certain phytopathogenic fungi such as *Fusarium* and *Alternaria* spp. are known to produce mycotoxins with insecticidal properties (Gupta et al., 1991; Abbas and Mulrooney, 1994; Logrieco et al., 1998). However, the significance of these interactions on the performance of root-feeding insects has not been determined.

### Insect Feeding Injury Facilitates Pathogen Infection

Significant attention has been focused on the use of insect feeding wounds as infection sites by fungal and oomycete pathogens; in some cases, the interaction is strong enough that the insect is viewed as part of the disease complex (Godfrey and Yeargan, 1987; Kalb et al., 1994). Typically, these instances involve larval coleopterans that feed on roots or, in leguminous plants, *Rhizobium* root nodules, and pathogens such as *Fusarium* Link spp. that are associated with root rot diseases. *Fusarium* spp. frequently infect undamaged roots directly, but are broadly classified as opportunistic pathogens that become problematic in plants stressed by adverse biotic or abiotic conditions (Dickason et al., 1968; Stutz et al., 1985; Kalb et al., 1994; Harveson et al., 2005). The clover root curculio, *Sitona hispidulus* F. (Coleoptera: Curculionidae), has a well-established association with root rot disease caused by *Fusarium* spp. in forage legumes (Dickason et al., 1968; Leath and Hower, 1993; Kalb et al., 1994). *S. hispidulus* larvae produce deep wounds on roots and *Rhizobium* nodules that serve as entry points for *Fusarium* spp., significantly increasing the incidence and severity of cortical and vascular decay in alfalfa (*Medicago sativa* L.) and clover (*Trifolium* L. spp.) (Leach et al., 1963; Thompson and Willis, 1967; Kalb et al., 1994). *Fusarium* spp. are the dominant pathogens associated with *S. hispidulus* damage, though fungi and oomycetes from several other genera including *Rhizoctonia, Phoma*, *Trichoderma*, *Pythium*, and *Verticillium* have also been isolated from feeding wounds (Godfrey and Yeargan, 1987; Leath and Hower, 1993).

The interaction between *S. hispidulus* and soilborne pathogens is not unique; several species of root-feeding insects are known to predispose their hosts to disease, and it is likely that many others have gone undetected. *Fusarium* root rot is also associated with the feeding activity of the clover root borer and the weevil *Calomycterus setarius* Roelofs (Coleoptera: Curculionidae) in red clover (Newton and Graham, 1960; Jin et al., 1992). The feeding activity of the western corn root worm (*Diabrotica virgifera virgifera* LeConte; Coleoptera: Chrysomelidae) strongly accelerates colonization of *Fusarium verticillioides* (Saccardo) Nirenberg in maize roots (Kurtz et al., 2010). Fungus gnat larvae (*Bradysia* spp.; Diptera: Sciaridae) promote infection by both *Fusarium* and *Pythium* spp. in soybean and alfalfa (Leath and Newton, 1969; Graham and McNeill, 1972). In sainfoin, suppression of *Sitona scissifrons* Say populations with insecticides reduced the incidence of root disease by half (Morrill et al., 1998), clearly demonstrating the importance of identifying root-feeding insects as risk factors for infection.

### Insects as Vectors of Fungal Phytopathogens

The close association of fungi and oomycetes with root injury caused by herbivores has led to the suggestion that some insects may further facilitate infection by transmitting pathogens to host plants. The role of insects as vectors of phytopathogens has been well-established. In comparison to viruses, phytoplasmas, and other pathogens that form highly specific relationships with insects, the transmission of fungal and oomycete pathogens by insects is usually adventitious (Agrios, 2008). Spores or mycelia adhering to or ingested by insects can be passively transported to uninfected plant tissues (Agrios, 2008). In aboveground systems, there are ample examples of insects that disseminate fungal pathogens. Many of these interactions involve accidental transmission, whereas others are more specialized. The fungus responsible for Dutch elm disease, for example, attracts the elm bark beetle *Hylurgopinus rufipes* Eich. (Coleoptera: Curculionidae) by inducing the upregulation of attractive sociochemicals in infected trees in a complex indirect interaction (McLeod et al., 2005). Attempts to link root-feeding insects to the spread of fungi and oomycetes are often tenuous, however, due to the limited mobility of subterranean insects and the difficulty of observing interactions in the rhizosphere. Accordingly, few insects have been confirmed as vectors of soilborne fungi and oomycetes. Given their close association, it is likely that fungi and oomycetes colonizing insect feeding wounds may also be physically transported to injured tissue as insects move between plants to feed. *Fusarium* spp. have been isolated from the head capsules of *S. hispidulus* larvae (Leath and Hower, 1993), and the introduction of *Fusarium avenaceum* to clover fields has been associated with the movements of clover root borers (Jin et al., 1992). There is incidental evidence that root borers vector other fungi such as *Kabatiella caulivora* (Kirch.) Karak and *Colletotrichum trifolii* Bain (Poos et al., 1955), and may ingest and excrete viable fungal spores (Leath et al., 1971). This relationship is particularly intriguing given the attraction of root borers to diseased roots (Leath and Byers, 1973), as this presumably would increase their exposure to phytopathogenic fungi and therefore their efficiency as a vector.

In a closed environment such as a commercial greenhouse, insect-facilitated pathogen dissemination could rapidly lead to large-scale infection and losses in yield. This may be particularly important for oomycete pathogens that are not capable of aerial dispersal, relying instead on belowground reproductive structures and the mechanical movement of water, soil, or infected plant material (Hyder et al., 2009; Braun et al., 2012). The transmission of oomycetes and fungi by larvae of common dipteran greenhouse pests such as fungus gnats (*Bradysia impatiens*), shore flies (*Scatella stagnalis* Fallén; Diptera: Ephydridae), and moth flies (*Psychoda* spp.; Diptera: Psychodidae) has received considerable attention as a potential risk factor for disease spread (Gardiner et al., 1990; El-Hamalawi, 2008a,b). Oospores, fungal spores, and other propagules can retain viability following passage through the digestive tract of larvae, and can then be transmitted to healthy plants (Goldberg and Stanghellini, 1990; El-Hamalawi, 2008a,b; Hyder et al., 2009; Braun et al., 2012). Interestingly, gut passage appears to benefit the pathogen in some cases: infectivity of oospores from one strain of *Pythium aphanidermatum* (Edson) Fitzp. and chlamydospores produced by the fungus *Thielaviopsis basicola* (Berk. & Broome) is increased following excretion (Stanghellini et al., 1999; Braun et al., 2012). However, the low mobility of larvae preclude them from moving between individual pots or benches within a greenhouse, thus transmission is only likely in seedling flats, hydroponic troughs, and other circumstances in which the pathogen can spread on its own (Braun et al., 2012). Retention of viable propagules from larval stages into the highly mobile adult stage would greatly increase the potential for pathogen dispersal. Transstadial transmission of *Pythium* spp., *T. basicola, Verticillium dahliae* Kleb, and *Fusarium* spp. occurs in shore flies, but is not evident in fungus gnats or moth flies (Goldberg and Stanghellini, 1990; El-Hamalawi, 2008a,b). It therefore seems unlikely that *B. impatiens* and *Psychoda* spp. are significant factors in the spread of soilborne pathogens in a greenhouse setting. Further investigation into the ability of *S. stagnalis* to retain oospores and fungal propagules into adulthood may clarify the role of this insect as a vector.

## Indirect Interactions

The role of plants in mediating interactions between insects and phytopathogens has become a subject of interest relatively recently, and it has rapidly become clear that plant responses to attack have important impacts on the population dynamics of both insects and phytopathogens. Our current understanding of plant-mediated insect–phytopathogen interactions is based mainly on aboveground interactions. Significant progress has also been made in defining interactions between above- and belowground herbivores and pathogens that are mediated by plant defense responses (see reviews by Hatcher, 1995; Van der Putten et al., 2001; Bezemer and van Dam, 2005; Stout et al., 2006). Plants similarly modify interactions between pathogens and insects in the rhizosphere, but the mechanisms behind these interactions are often poorly understood. Root exudates such as ions, oxygen, water, enzymes, mucilage, and primary and secondary metabolites accumulate in the rhizosphere and can have important roles in plant biological processes, including defense against pathogens and insects (Bais et al., 2006). Sufficient data to draw definitive conclusions is lacking, but evidence suggests that root exudates may also play an important role in mediating interactions between insects and phytopathogens in soil. Stutz et al. (1985) found that wounded alfalfa and red clover roots induced the production of distributive hyphae rather than chlamydospores in some species of *Fusarium*, accelerating fungal penetration and colonization near, but not in, injured tissue. Exudates from injured roots may have augmented the nutritional status of the rhizosphere, thus favoring pathogen growth (Stutz et al., 1985). This is consistent with reports that the weevil *Diaprepes abbreviatus* L. (Coleoptera: Curculionidae) promotes infection of citrus roots by *Phytophthora* spp. in tissues spatially separated from feeding wounds (Rogers et al., 1996; Graham et al., 2003). Increased decay in tissues distal to weevil damage was attributed to sugars and other nutritional compounds leaking into the rhizosphere from damaged cells (Graham et al., 2003). In contrast, herbivory of geranium roots by fungus gnat larvae increased seedling resistance to infection by *Pythium aphanidermatum*, possibly due to an induced defense response (Braun et al., 2009).

Further investigation of how root chemical signals influence the population dynamics of multiple organisms will be critical to our understanding of plant defense and rhizosphere ecology. Plant-mediated interactions between belowground insects and phytopathogens are undoubtedly complex, and our picture of how root exudates influence these interactions is largely incomplete. Chemical signals from roots attract and repel insects and pathogens (Bais et al., 2006), alter nutritional quality (Stutz et al., 1985; Graham et al., 2003), and attract natural enemies of herbivores in sophisticated tritrophic interactions similar to those occurring aboveground (Rasmann et al., 2005). Future research is required to reveal how root exudates influence the population dynamics of insects, phytopathogenic fungi and oomycetes. This will be vital in increasing our understanding of how plants defend themselves against diverse organisms in the rhizosphere.

## Utilizing Synergistic Insect–Fungal Interactions in Biological Control

Synergistic interactions between herbivores and phytopathogens can be problematic in agricultural crops, but have potential for use in biocontrol. Insects and fungi are the primary organisms used in weed biocontrol, but are rarely used in combination (Hatcher and Paul, 2001; Caesar et al., 2010). Synergisms between root-feeding insects and soilborne pathogens have been identified in several species of rangeland weeds. Spotted and diffuse knapweed, *Centaurea maculosa* Lam. and *C. diffusa* Lam., are pervasive across the western United States and Canada (Caesar et al., 2002). Within their native Eurasian range, populations are apparently kept in check by a combination of root herbivory and fungal infection. Several highly virulent strains of *Fusarium* spp. are associated with the feeding wounds of *Cyphocleonus* (Coleoptera: Curculionidae) and *Agapeta* spp. (Lepidoptera: Cochylidae) in their larval stage (Caesar et al., 2002). Likewise, populations of *Fusarium* spp. associated with leafy spurge (*Euphorbia esula/virgata* L.) increase in density around roots attacked by *Aphthona* spp. (Coleoptera: Chrysomelidae), *Chamaesphecia* spp. (Lepidoptera: Sesiidae), and *Oberea erythrocephala* (Schrank) (Coleoptera: Cerambycidae) (Caesar, 2003). Greenhouse experiments showed that *E. esula/virgata* exposed to combinations of *Rhizoctonia solani* Kühn, *F. oxysporum* (Schlect) Snyd. & Hans., and *Aphthona* spp. had higher disease levels than any single inoculation (Caesar, 2003). The invasive perennial weed *Lepidium draba* L. ssp. *draba* sp. [ = Cardaria draba (L.) Desv.] is associated with the gall-forming weevil *Ceutorhynchus assimilis* Paykull (Coleoptera: Curculionidae) (Fumanal et al., 2004; Caesar et al., 2010). Some populations within this species demonstrate a high level of host-specificity to *L. draba* as larvae (Fumanal et al., 2004). *Ceutorhynchus assimilis* galls are frequently colonized by pathogenic *Rhizoctonia* and *Fusarium* spp., suggesting that galling promotes fungal infection (Caesar et al., 2010). Many of the insect and pathogen species associated with rangeland weeds also have narrow host ranges, making them suitable for consideration as biocontrol agents (Caesar et al., 1999, 2002, 2010). Synergistic interactions between insects and plant pathogens can enhance weed management and provide an effective alternative to herbicides in multiple plant species. Despite this, utilization of these interactions in weed biocontrol remains rare, thus emphasizing the importance of identifying the impact of insect–pathogen interactions on plant populations.

## Summary

Root-feeding insects are closely associated with the soilborne microorganisms that colonize their mutual plant host. Direct and indirect interactions between insects and pathogenic fungi or oomycetes can increase the incidence and severity of host injury, influencing plant performance and mortality. Tripartite interactions have been identified in several agroecosystems, but it is likely that many more have gone undetected. Belowground interactions are difficult to observe, and have largely been ignored despite the potential impacts of root damage on overall plant functioning. Soilborne fungi and oomycete pathogens may interact directly with root-feeding insects by colonizing injured plant tissue. Some insects vector soilborne pathogens via internal or external transport, which may be of particular importance in commercial greenhouses and other closed environments. Plant responses to attack appear to be important factors in defining belowground insect–fungi interactions, but our mechanistic understanding of these interactions remains limited. Identifying and understanding tripartite interactions between plants, insects and pathogens will therefore be an important step in furthering our understanding of plant defense responses to belowground threats. An emphasis on integrating entomological and pathological research will allow for more accurate predictions of pest-related crop damage, and will guide effective monitoring and crop protection strategies. Finally, interactions between insects and microorganisms have the potential for use in biocontrol. Synergism between herbivores and pathogens of rangeland weeds suggest that suppression of invasive plant species can be increased by coordinating the release of multiple antagonists. Whether they appear as beneficial organisms or damaging pests, root associated insects, fungi and oomycetes have important consequences for plant health, and the interactions between these organisms are worthy of further study.

## Author Contributions

TW collected literature and wrote the paper. SC and HC provided revisions, and all three authors approved this mini review for publication.

## Conflict of Interest Statement

The authors declare that the research was conducted in the absence of any commercial or financial relationships that could be construed as a potential conflict of interest. The reviewer SS and handling Editor declared their shared affiliation.
